# Supplementary material for: Integrating all-optical switching with spintronics
Source: Nat Commun. 2019 Jan 10;10:110. doi: 10.1038/s41467-018-08062-4 (PMC6328538; doi:10.1038/s41467-018-08062-4)
Supplement: Supplementary file 1 — Supplementary Information [file 41467_2018_8062_MOESM1_ESM.pdf]

Supplementary Information

Integrating all-optical switching with spintronics

Lalieu et al.

Supplementary Note 1: AOS as a function of the overlap between laser spot and Hall cross

The result presented in Fig. 1(b) of the main Article shows full AOS in the Hall cross. This was measured with the centre of the laser spot aligned to the centre of the Hall cross. As was mentioned, the laser spot was larger than the cross, meaning that the area of the Pt/Co/Gd wire that is exposed (and switched) by the laser pulse is larger than the region probed by the Hall cross. It is known that depending on the laser fluence, a multidomain state can form at the centre of the (Gaussian shaped) laser spot, in which case only AOS is observed in an outer rim of the excited area[1]. In this section, it is verified that a single homogeneous domain was written in the Pt/Co/Gd wire by the laser pulse.

In order to check that a homogeneous domain is written by the laser pulse, the measurement performed in Fig. 1 of the main Article was repeated for different alignments of the laser spot with respect to the Hall cross. At each new alignment, the magnetization in the wire was first saturated using an externally applied field, whereafter the field was turned off and the Hall cross was exposed to a single laser pulse. When there is (partial) overlap between the Hall cross and the center area of the laser spot where the fluence $F(x, y)$ is above the AOS threshold fluence F_0, the magnetization in the Hall cross will be switched, which is recorded by a step in the AHE signal (similar as shown in Fig. 1(b) of the main Article). The size of the AHE step is proportional to the area of the Hall cross that is switched by the laser.

Supplementary Figure 1 shows the normalized AHE step size as a function of x and y position, where $(x, y) = (0, 0)$ corresponds to the center of the (Gaussian) laser spot being aligned to the center of the Hall cross. The x (black) and y (red) scans are performed with $y = 0$ and $x = 0$, respectively, and each data point is an average of 7-8 subsequent measurements. When the laser spot is sufficiently far away from the Hall cross, i.e., for $|x|, |y| > 15$ μm, the AHE step size is zero, meaning that there is no overlap between the Hall cross and the laser spot (or at least no sufficient overlap). Moving the laser spot closer to the Hall cross, i.e. decreasing $|x|$ or $|y|$, the AHE step size increases towards saturation at $|x|, |y| \approx 5$ μm. The increase in AHE step size corresponds to the center part of the laser spot, where $F(x, y) \geq F_0$, moving into the Hall cross area. This is illustrated by the left and right cartoons in the figure in which the laser spot area with $F(x, y) \geq F_0$ (red dotted circle) overlaps $\approx 30\%$ of the Hall cross area. For $|x|, |y| < 5$ μm the AHE step size is constant

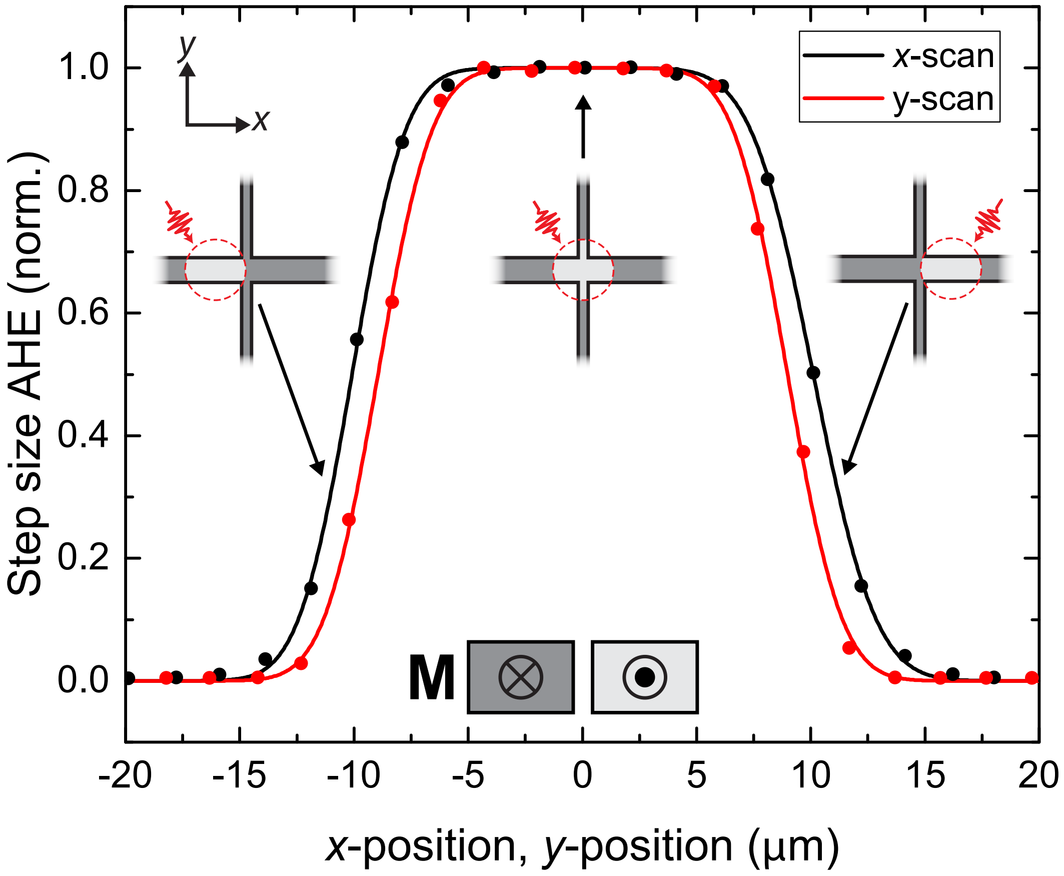

Supplementary Figure 1. **AOS as a function of the overlap between laser spot and Hall cross.** Normalized step size of the AHE signal as a function of the x and y position. The step size in the AHE signal measured across the Hall cross is a measure of the area of the Hall cross that is switched by the laser pulse. $(x, y) = (0, 0)$ corresponds to the center of the laser spot being aligned to the center of the Hall cross.

and equal to saturation. The presence of these plateaus in both x and y scans demonstrates that for the full area of the laser spot where $F(x, y) \geq F_0$ there is full AOS. In other words, this means that indeed a homogeneous domain was written in the Pt/Co/Gd wire by the laser pulse. As a side note, the full width at half maximum of the curves are equal to the size of the written magnetic domain along the x and y directions, showing a domain size of ≈ 20 μm and a slightly elliptically shaped laser spot, which was verified using wide field Kerr microscopy on a full-sheet sample.

Lastly, it is known that when the pulse energy is sufficiently increased, the fluence at the center of the laser spot can be increased to a value above a second threshold fluence. At

this fluence the lattice temperature is heated above the Curie temperature, resulting in the formation of a multidomain state on cool down. Such a multidomain state at the center of the written domain would show up as a dip in the AHE step size around $(x, y) = (0, 0)$ in the measurement presented in Supplementary Figure 1. Such a dip was indeed observed when repeating the measurement of Supplementary Figure 1 with increasing pulse energy (not shown), verifying that inhomogineties in the written domain can indeed be measured, and thus confirming that for the laser pulses used in Supplementary Figure 1 and the main Article a homogeneous domain was written.

Supplementary Note 2: DW pinning and Ga^{+} irradiation

In the main Article it was mentioned that the legs of some of the Hall crosses were irradiated with Ga^{+} ions in order to prevent pinning at the entrance of the cross. The pinning of the DW at the entrance of a non-irradiated Hall cross in a typical on-the-fly AOS measurement as performed in the main Article is demonstrated in the measurement discussed in Supplementary Note 3 [Supplementary Figure 3(c)]. A visual presentation using a Kerr microscope is presented in the top row of Supplementary Figure 2. In this figure a down domain (dark) in an otherwise up (light) magnetized Pt/Co/Gd wire is located between two Hall crosses. Using three current pulses of alternating direction (see figure) it can be seen that the domain tries to move along the current direction, but gets pinned at the entrance of the cross it is moving towards. Moreover, it can be seen that the end points of the DW get pinned at the start of the legs, while the center of the DW gets pushed into the cross, which will be visible in the AHE signal (as demonstrated in Supplementary Note 3).

The pinning of the DW at the entrance of the Hall cross happens due to the fact that the DW length has to increase in order to pass through the cross[2]. One way to overcome this problem, while still being able to use the legs for the AHE measurement, is to magnetically 'cut-off' the legs using a technique called magnetic etching[3]. In this technique a magnetic sample with perpendicular magnetic anisotropy (PMA) is exposed to Ga^{+} ion irradiation with a relatively high dose (40 μC cm^{-2} in this work), which is enough to destroy the magnetic anisotropy, but not enough to physically remove a significant amount of material. Using this technique the PMA in (part of) the legs is destroyed, causing it to become in-plane magnetized (or even paramagnetic), while the legs keep their conductive properties

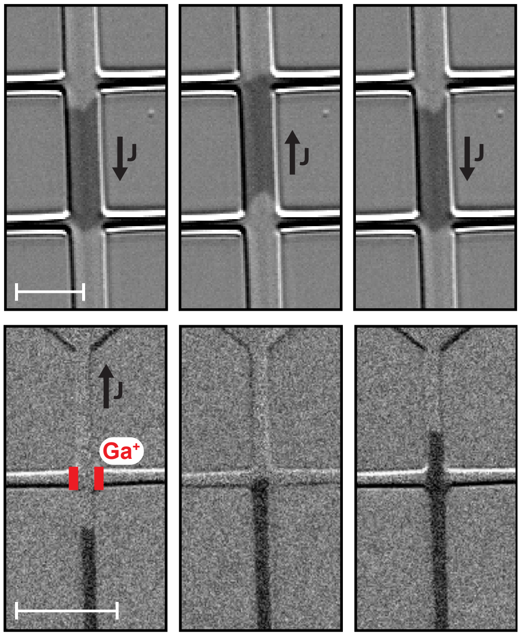

Supplementary Figure 2. **DW pinning at a Hall cross and the effect of Ga$^+$ ion irradiation.** Top row: A Pt/Co/Gd wire with a down domain (dark) in an otherwise up (light) magnetized wire that is located between two Hall crosses. The three figures show the domain after three current pulses of alternating direction (direction indicated in figures). Bottom row: A Hall cross on a 2 μm Pt/Co/Gd wire with a DW initially located below the cross. The red squares in the left figure represent the regions that are exposed to the Ga$^+$ ions. The figures show three snapshots of the DW moving through the wire by the SHE using a DC current (direction indicated in left figure). The scale bars in the figures corresponds to 10 μm.

needed for the AHE measurement. In this way the AHE measurement can still be performed to measure the magnetization in the Hall cross area, while the legs become invisible for the DW. As a result, the DW will not be pinned at the entrance of the Hall cross since it does no longer need to increase in length when passing through the cross.

The effect of the Ga$^+$ ion irradiation on the DW propagation through the Hall cross is verified in the bottom row of Supplementary Figure 2. In these figures a Hall cross on a 2 μm Pt/Co/Gd wire is shown, where the red squares in the left figure represent the regions that are exposed to the Ga$^+$ ions. Starting with a DW below the Hall cross in the left figure, three snapshots are shown of the DW moving through the wire by the SHE using a DC current. It is seen that with the legs being magnetically cut-off, the DW indeed is able to move past the Hall cross without getting pinned.

Supplementary Note 3: On-the-fly AOS with different laser spot to (non-irradiated) Hall cross alignments

A proof-of-concept measurement was presented in Fig. 2 of the main Article, demonstrating on-the-fly single-pulse AOS and simultaneous SHE driven motion of magnetic domains in a single racetrack. In that measurement, the laser spot was aligned to the right side of the first Hall cross to prevent pinning of the DW's (see Fig. 2(a) of the main Article), while a small overlap between laser pulse and the first Hall cross was maintained in order to verify if and when a domain was written. In this section, two similar measurement are presented; (i) with the laser spot centered at the center of the first Hall cross, clearly demonstrating the pinning of the DW at the entrance of the non-irradiated Hall cross, and (ii) with the laser spot aligned completely in between the two Hall crosses.

An illustration of the on-the-fly AOS measurement performed with the laser spot aligned to the center of the first Hall cross is shown in Supplementary Figure 3(a). The expected magnetic behavior is as follows; (I) With the magnetization in the wire initially saturated, the first (left) cross is exposed to a single laser pulse (red dotted circle), writing a magnetic domain that is larger than the Hall cross, thus creating a DW at either side of the cross. (II) Due to the SHE originating from the DC current that is continuously sent through the wire, both DW's (and thereby the domain) move along the current direction towards the second Hall cross. The DW written in between the two Hall crosses will reach the second cross, where it can pass the cross since its legs are magnetically cut-off using Ga^{+} ion irradiation. The legs of the first Hall cross, however, have not been irradiated. Therefore, the DW written to the left of the first cross will get pinned at the entrance of the cross. The center of the DW is pushed into the cross by the SHE, and will be measurable by the AHE. (III) The next laser pulse will toggle the magnetization in the exposed area, creating two new DW's and reversing the polarity of the DW located in the first Hall cross. (IIII) The newly created DW's are moved along the wire by the SHE and annihilate with the previously written DW's, leaving the wire in the saturated state. This process will then repeat itself at the next laser pulse.

First, a test measurement is performed without a DC current being sent through the wire, and using a repetition rate of 0.1 Hz to clearly see the effect of the single laser pulses. The result is presented in Supplementary Figure 3(b), showing the normalized AHE signal of both crosses as a function of time. The top graph shows the AHE signal of the first cross,

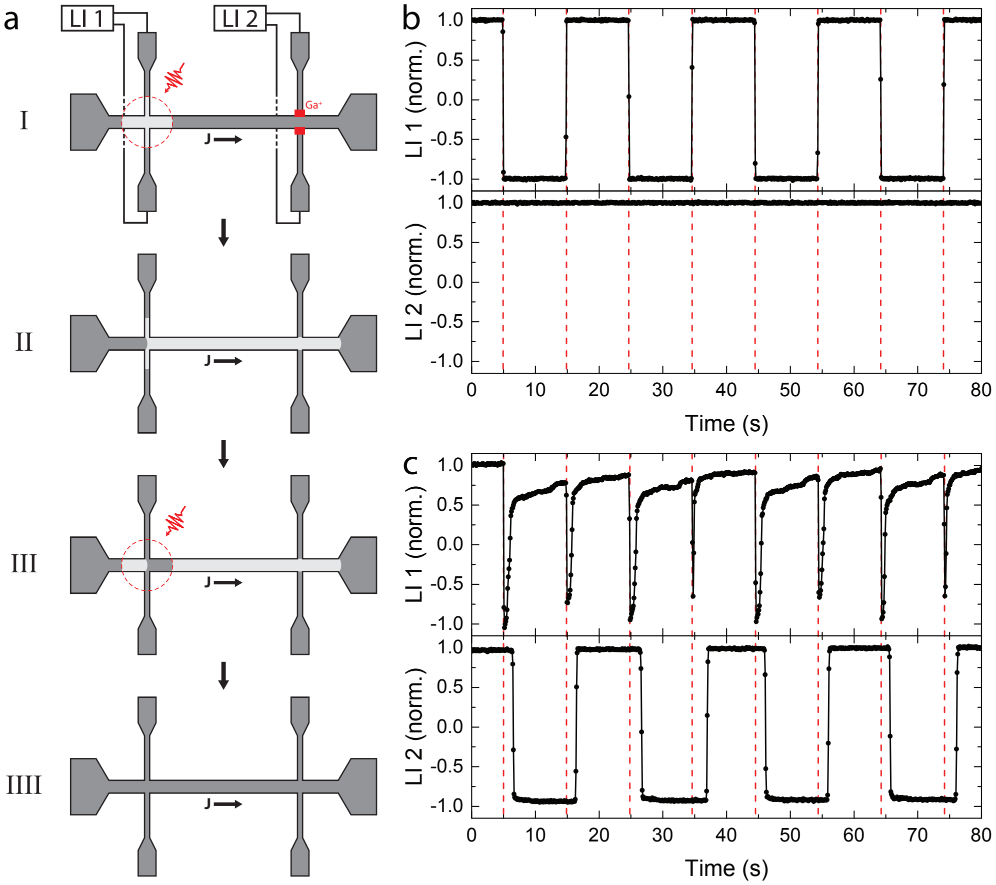

Supplementary Figure 3. **On-the-fly AOS with the laser spot centered on the non-irradiated Hall cross.** (a) Illustration of the on-the-fly AOS measurement performed with the laser spot aligned to the center of the first Hall cross. The AHE signal in the Hall crosses is measured using lock-in amplifiers LI 1 and LI 2. The red dotted circle illustrates the region exposed by the laser pulse, and the red blocks indicate the regions exposed to Ga^+ ion irradiation. Figures I to IIII show different snapshots of the magnetization in the wire during a two-pulse cycle. (b,c) Measurement of the normalized AHE signal as a function of time in the first (top) and second (bottom) Hall cross while at the same time the first Hall cross is exposed to a train of linearly polarized laser pulses ($\approx$ 100 fs) at a laser-pulse repetition rate of 0.1 Hz. The measurement presented in (b) was performed without any current sent through the wire, while a current of +5.5 mA was sent through the wire during the measurement presented in (c).

demonstrating clear and full single-pulse AOS of the magnetization in this cross, similar as shown in Fig. 1(b) of the main Article. The bottom graph shows the AHE signal of the second Hall cross. As can be seen by the constant AHE signal at the initial saturation

value, there is no effect of the laser pulses on the magnetization in the second cross, which is expected since there is no current sent through the wire, and thus no DW motion.

Supplementary Figure 3(c) shows the result of a measurement with a DC current of +5.5 mA sent through the wire. Looking at the AHE signal of the first Hall cross (top graph), the signal looks much different than the signal measured without a DC current [Supplementary Figure 3(b)]. Before explaining the observed behavior in more detail, it is noted that the times at which the magnetic domains are written at the first Hall cross are still clearly visible by the sudden steps in the AHE signal (red dotted lines). Looking at the AHE signal in the second Hall cross (bottom graph), it can be seen that shortly after the domains are written, the magnetization in the second cross switches its direction, toggling up and down after each subsequent laser pulse. The toggling behavior corresponds to the DW that is written to the right of the first Hall cross that is transported along the wire by the SHE and passes through the second Hall cross. As shown in Supplementary Figure 3(a), this DW alternates between an up-down and down-up DW on subsequent laser pulses, leaving the magnetization in the cross in the up and down state after the DW has passed, respectively.

Coming back to the AHE signal measured in the first Hall cross [top graph Supplementary Figure 3(c)], there are two observations that can be made. Firstly, the step in the AHE signal varies in size and has the same sign for every laser pulse. Secondly, the AHE signal after every even number of pulses is not equal to the initial saturation value of +1. Both observations will be discussed in the following.

It can be seen that the very first laser pulse switches the magnetization from the initial saturated up (+1) direction to the down (−1) direction. In contrast to the case without the DC current, the AHE signal immediately rises again towards $\approx +0.5$. This corresponds to the DW written to the left of the first Hall cross being transported along the wire by the SHE and getting pinned at the entrance of the cross (Supplementary Figure 3(a).II). The center of the DW is being pushed inside the cross by the SHE, causing about 75% of the Hall cross area being switched back to the initial saturation direction. After this initial (fast) response, the center of the DW keeps getting pushed further inside the cross at a slower rate, causing a further (slow) increase of the AHE signal. When the second pulse hits the sample, most of the Hall cross area ($\approx$ 85%) is switched back to the initial saturation direction (Supplementary Figure 3(a).III). Therefore, the second step in the AHE signal has the same sign as for the first pulse, however, the size of the step is smaller since it only corresponds to the net switched magnetization. This measurement clearly demonstrates the

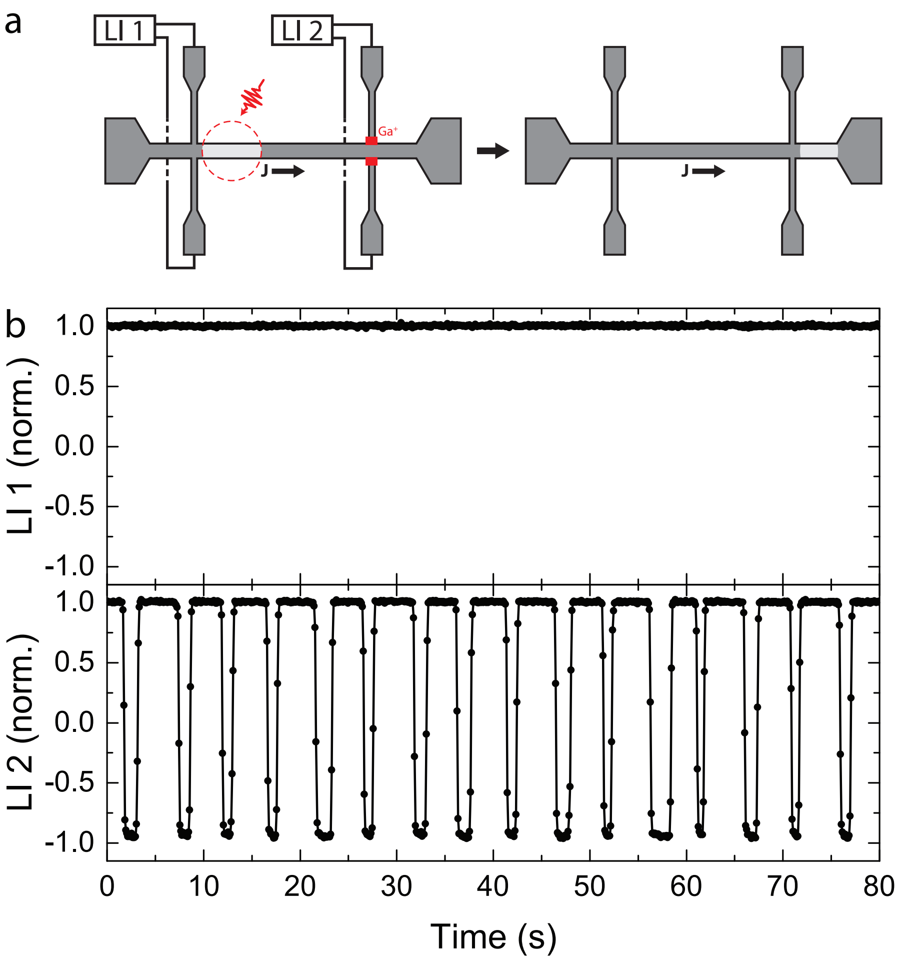

Supplementary Figure 4. **On-the-fly AOS with the laser spot aligned in between the two Hall crosses.** (a) Illustration of the on-the-fly AOS measurement performed with the laser spot aligned in between the two Hall crosses of the Pt/Co/Gd wire. The AHE signal in the Hall crosses is measured using lock-in amplifiers LI 1 and LI 2. The red dotted circle illustrates the region exposed by the laser pulse, and the red blocks indicate the regions exposed to Ga^+ ion irradiation. (b) Measurement of the (normalized) AHE signal as a function of time in the first (top) and second (bottom) Hall cross while at the same time the wire is exposed to a train of linearly polarized laser pulses ($\approx$ 100 fs) at a laser-pulse repetition rate of 0.2 Hz and a DC current of +5.5 mA is sent through the wire.

pinning of the DW at the entrance of the Hall cross when the legs are not magnetically cut-off using Ga^+ ion irradiation.

Taking a closer look at the AHE signal in the first Hall cross just before the third pulse arrives, it can be seen that the signal is not back to the saturation value of +1, but is close to

+0.9 which corresponds to 95% of the first Hall cross having its magnetization up. Looking back at Supplementary Figure 3(a), it was expected that the DW written on the left side of the cross by the second pulse (or every even amount of pulses) would annihilate with the DW pinned in the cross (written by the previous pulse), leaving the Hall cross in the initial saturated state. This discrepancy is believed to be the result of the center of the DW that is being pushed into the cross not reaching a steady position before the next laser pulse arrives. This could result in the formation of thin rings with alternating magnetization direction in the first Hall cross that slowly move along with the current. The presence of these rings cause the AHE signal not to reach the saturation value of +1 after every even amount of pulses.

Lastly, the same on-the-fly AOS measurement is performed, but now with the laser spot aligned completely in between the two Hall crosses, as shown in Supplementary Figure 4(a), and a laser-pulse repetition rate of 0.2 Hz. The AHE signal of both Hall crosses as a function of time is shown in Supplementary Figure 4(b). The magnetic domains are written to the right side of the first Hall cross, and they are transported along the current direction. This means that none of the domains or DW's reach the first Hall cross, resulting in the observed constant saturation value of the AHE signal in the first Hall cross (top graph). Looking at the AHE signal of the second Hall cross (bottom graph), it can be seen that all the optically written domains pass the second cross, as was also demonstrated in the measurement presented in Fig. 2(b) of the main Article. Also seen in both measurements is the variation in the width of the domains when they pass the second cross (proportional to the time between down and up switch in the AHE signal). This shows that the variation in domain width measured in Fig. 2(b) of the main Article is not dominated by the slight overlap between laser spot and first Hall cross, but is more likely to be the result of random pinning along the wire, as was already stated in the main Article.

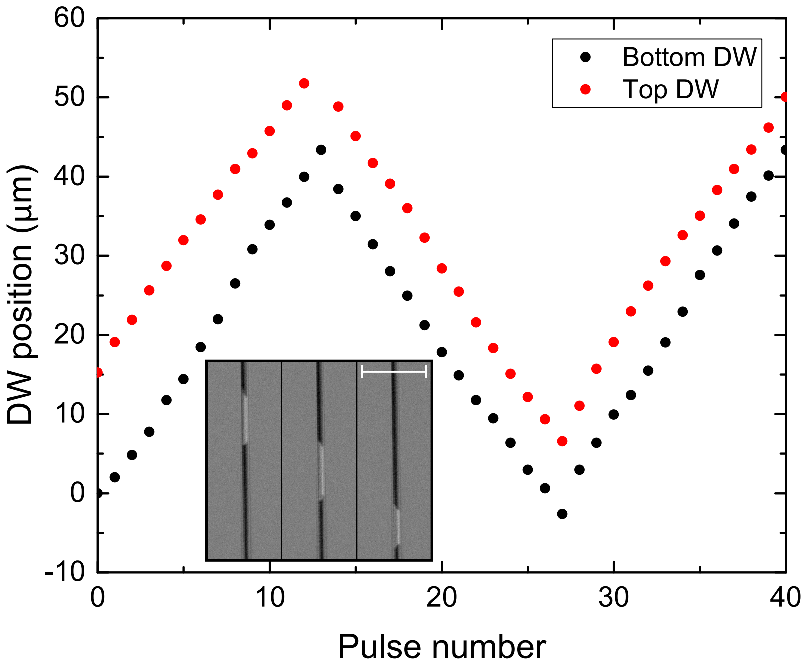

Supplementary Figure 5. **SHE driven domain wall velocity measurement.** The position of both domain walls enclosing a magnetic domain in a 2 μm wide Pt/Co/Gd racetrack is plotted as a function of the number of current pulses passed through the racetrack . The current pulses have a duration of 0.5 μs and a current density of $3.8 \cdot 10^{11}$ A m^{-2}. When the domain reached either end of the racetrack, the current direction is reversed. The inset shows three snapshots of the magnetization in the racetrack during the measurement, taken using a Kerr microscope. The scale bar in the inset figure corresponds to 20 μm.

Supplementary Note 4: Pulsed domain wall velocity measurement (≈ 7 m s^{-1}) in Pt/Co/Gd racetrack

In the main Article, it was mentioned that the SHE driven DW velocity in the measurement presented in the inset of Fig. 2(b) of the main Article was equal to ≈ 7 m s^{-1}. In this section, the corresponding measurement of the DW velocity is presented.

The measurement is performed on a 2 μm wide Pt/Co/Gd racetrack. Before the measurement, the magnetization in the racetrack is saturated in the down direction (black), and an up magnetized domain (white) is inserted using an external magnetic field, see the inset of Supplementary Figure 5. Next, the external magnetic field is turned off, and a purely SHE induced DW motion is achieved using a train of current pulses with a duration of 0.5 μs and a current density of $3.8 \cdot 10^{11}$ A m^{-2} (using a homogeneous current distribution

throughout the stack in the current density calculation). After each current pulse, an image of the magnetization in the racetrack is taken using a Kerr microscope in order to determine the position of the two DW's enclosing the magnetic domain. When the domain reached either end of the racetrack, the current direction is reversed.

The position of the two DW's after each current pulse is plotted in Supplementary Figure 5, in which the position is plotted relative to the initial position of the bottom DW. The average DW velocity is calculated by measuring the traveled distance for each pulse and dividing it by the current pulse duration. The obtained average DW velocity is 7 ± 1 m s^{-1}. As can be seen, the DW's slowly creep towards each other. As was already discussed in the main Article, this is attributed to random pinning of the DW's, which move in the pinning dominated creep regime.

Supplementary References

1 Hadri, M. E. et al. Two types of all-optical magnetization switching mechanisms using femtosecond laser pulses. *Phys. Rev. B* **94**, 064412 (2016).

2 Ravelosona, D., Lacour, D., Katine, J., Terris, B. & Chappert, C. Nanometer scale observation of high efficiency thermally assisted current-driven domain wall depinning. *Phys. Rev. Lett.* **95**, 117203 (2005).

3 Franken, J., Swagten, H. & Koopmans, B. Shift registers based on magnetic domain wall ratchets with perpendicular anisotropy. *Nat. Nanotechnol.* **7**, 499–503 (2012).
